# A Severe Clinical Case of *Ehrlichia canis* and *Toxoplasma gondii* in a Dog (With the First Morphological Detection of Tachyzoites in Peripheral Blood)

**DOI:** 10.1002/vms3.70380

**Published:** 2025-05-06

**Authors:** Ioana Sandu, Georgiana Deak, Mihai Turcitu, Peter James O'Brien, Viorica Mircean

**Affiliations:** ^1^ Department of Parasitology ‐ Parasitic Diseases and Animal Biology University of Agronomic Sciences and Veterinary Medicine of Bucharest Bucharest Romania; ^2^ Pet Stuff Veterinary Hospital Bucharest Bucharest Romania; ^3^ Department of Parasitology and Parasitic Diseases University of Agricultural Sciences and Veterinary Medicine of Cluj‐Napoca Cluj‐Napoca Romania; ^4^ Omnivet Impex SRL Bucharest Romania; ^5^ Department of Pathobiology University College Dublin Dublin Ireland; ^6^ Dermatology Clinic University of Agricultural Sciences and Veterinary Medicine of Cluj‐Napoca Cluj‐Napoca Romania

**Keywords:** diagnosis, dog, *Ehrlichia canis*, Romania, *Toxoplasma gondii*

## Abstract

Vector‐borne pathogens (VBPs) are frequently identified in domestic dogs, particularly in endemic areas or in individuals lacking protection from anti‐feeding products. Diagnostic procedures in veterinary clinics for symptomatic animals primarily include rapid serological assays; however, more definitive, albeit time‐intensive tests typically require well‐equipped laboratory facilities.

A 5‐year‐old female mixed‐breed dog was presented to a veterinary clinic exhibiting neurological abnormalities, dyspnoea, lethargy and anorexia. Multiple ticks, predominantly of the genera *Rhipicephalus* and *Dermacentor*, were observed attached to the animal's integument. A blood smear examination revealed tachyzoites morphologically consistent with Toxoplasma spp. and intracellular morulae indicative of *Ehrlichia canis* infections. These findings were subsequently confirmed via polymerase chain reaction (PCR).

This report documents a rare and fatal canine co‐infection of ehrlichiosis and toxoplasmosis, initially diagnosed through microscopic examination and serology, with definitive confirmation achieved through PCR analysis.

## Graphical abstract

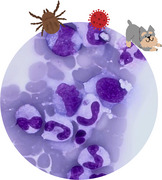

## 1 Background

**VBPs** are common in dogs, especially in individuals not protected by specific anti‐feeding products (Migliore et al. 2017; Manev 2020; Miró et al. 2022). Among these pathogens, *Ehrlichia canis* is a gram‐negative bacterium primarily transmitted by *Rhipicephalus sanguineus*, commonly known as the brown dog tick (Greene and Sykes 2023). Clinical signs of acute ehrlichiosis include pale mucous membranes caused by anaemia, epistaxis, petechiae, ecchymoses, prolonged bleeding during estrus, haematuria or melena (Mylonakis et al. 2019). These symptoms are typically associated with thrombocytopenia, thrombocytopathy or vasculitis (Sainz et al. 2015). Ocular manifestations are also seen in canine monocytic ehrlichiosis (CME) and may include anterior uveitis, corneal opacity, hyphaema, retinal vessel tortuosity, chorioretinal lesions, subretinal haemorrhage, retinal detachment and blindness. Neurological signs are less common and are generally secondary to meningitis (Sainz et al. 2015). In Romania, the prevalence of *Ehrlichia canis* in the canine population has been reported to range from 2.1% to 11.1% (Mircean et al. 2012; Miró et al. 2022). At present, a single clinical case of CME was previously documented in Timiș County (Morar et al. 2015). The diagnosis of the most important vector‐borne diseases in dogs, including ehrlichiosis, is commonly made in clinics using rapid serological tests (SNAP 4Dx Plus, SNAPLeishDx; IDEXX Laboratories) which proves only past exposure. In acute cases, diagnosis can also be supported through blood smear evaluation, including buffy coat analysis, cytological examination via fine needle aspiration (FNA) of lymph nodes and spleen, as well as PCR testing from blood or FNA samples (Sainz et al. 2015). Various serological techniques have been developed for diagnosing CME and are widely used as effective screening and diagnostic tools. The indirect immunofluorescence antibody (IFA) test for detecting anti‐*Ehrlichia canis* IgG antibodies is considered the serological gold standard, reflecting prior exposure to *E. canis*. However, IgM is not viewed as a reliable marker due to the inconsistent and often absent IgM response during the course of infection (Harrus and Waner 2011). To identify acute infections, it is recommended to perform two IFA tests spaced 7–14 days apart; a fourfold rise in antibody titers between tests suggests an active infection. Notably, anti‐ehrlichial IgG antibodies may persist for several months or even years following treatment and clearance of the organism (Mylonakis et al. 2019).

Toxoplasmosis, caused by the coccidian protozoan *Toxoplasma gondii* (phylum Apicomplexa, family Sarcocystidae), is a disease that affects a wide range of animal species and has a global distribution, with cats serving as the definitive hosts (Burkhard 2016). Transmission occurs via three primary routes: oral ingestion of oocysts from contaminated feline faeces, consumption of undercooked meat or organs containing cysts and transplacental transmission when the infection is acquired during pregnancy (Al‐Qassab et al. 2009; Calero‐Bernal and Gennari 2019). Reactivation of latent *T. gondii* infections primarily impacts the central nervous system (CNS), occasionally the lungs, and rarely other organs (Borges‐Silva et al. 2021; Blaga et al. 2023). Clinical disease is most common in immunosuppressed individuals (Patitucci et al. 1997; Barrs et al. 2006; Pepper et al. 2019). Dogs are intermediate hosts for *T. gondii* but generally show low morbidity and mortality and infrequently a primary disease that is linked to immunosuppression (Swinger et al. 2009; Al‐Qassab et al. 2009; Langoni et al. 2012). However, reactivation and triggering of a chronic form are possible in immunosuppression caused by various factors (Oliveira et al. 2014; Calero‐Bernal and Gennari 2019; Dini et al. 2024). Some documented manifestations of canine toxoplasmosis include neurological manifestations as well as noise sensitivity, myositis, various ocular conditions, and cutaneous forms (Webb et al. 2005; Papini et al. 2009; Hoffmann et al. 2012; Vallone et al. 2018). The diagnosis of canine toxoplasmosis poses significant challenges due to nonspecific clinical signs and the inherent limitations of diagnostic methods, including serological assays (such as ELISA or IFAT for IgM and IgG antibodies), PCR testing of cerebrospinal fluid (CSF), bronchoalveolar lavage (BAL) or blood samples, and microscopic identification of *Toxoplasma gondii* tachyzoites or bradyzoites via cytology or histopathology (Calero‐Bernal and Gennari 2019).

The present paper aimed to describe in detail a lethal co‐occurrence of clinical infection of canine toxoplasmosis and acute monocytic ehrlichiosis in a dog.

### 1.1 Case Presentation

A 5‐year‐old female mixed‐breed dog was presented to a veterinary emergency service in Bucharest, Romania, with clinical signs of altered mentation, dyspnoea, lethargy, and anorexia. The owner noted a gradual weight loss over the past 4 weeks, with the dog's weight declining from 11 to 7 kg, having a low (3/9) body condition score (BCS). The dog primarily resided in a yard in a rural area of Alexandria, Teleorman County, southern Romania, with free environmental access. At the time of presentation, the dog had no documented medical history and no veterinary treatments were done.

The clinical physical examination revealed tachypnoea, yellow mucous membranes, muscle weakness, petechial haemorrhages on the skin, primarily on the abdomen and ventral thorax, and purulent ocular and nasal discharge. Numerous ectoparasites, predominantly belonging to the genera *Rhipicephalus* spp. and *Dermacentor* spp., were identified affixed to the dog's skin. Physical examination further revealed generalized enlargement of the superficial lymph nodes upon palpation. The dog was mildly hypothermic (37.1°C), markedly hypotensive, exhibiting a systolic blood pressure of 40 mmHg (normal reference range for dogs: 90–120 mmHg, measured via Doppler), and moderately hypoglycemic, with a blood glucose level of 52 mg/dL (normal reference range for dogs: 70–120 mg/dL). Because of the muscle weakness and the petechial haemorrhages, a neurological evaluation was done, showing altered mentation (suggesting CNS involvement), a deficit in the menace response (indicating possible forebrain dysfunction), tetraparesis with intact spinal reflexes and proprioception (suggesting upper motor neuron involvement), and difficulty in rising and standing. These findings pointed to a multifocal neurological localization, with both central and peripheral components likely affected. At this point, the differential diagnosis included vectorial diseases and infectious pneumonia (viral, bacterial or parasitic). A peripheral blood sample was collected and tested for *Anaplasma* spp., *Leishmania* spp., *Ehrlichia* spp., and *Dirofilaria immitis* using a commercial ELISA method (SNAP LeishDx; IDEXX Laboratories), and all results yielded negative for these agents. In addition, because the dog did not receive any vaccines, a commercial CDV antigen test (Antigen Rapid CDV Ag Test Kit, BioNote, Hwaseong, Korea) was performed using conjunctival secretions, with negative results.

**FIGURE 1:**
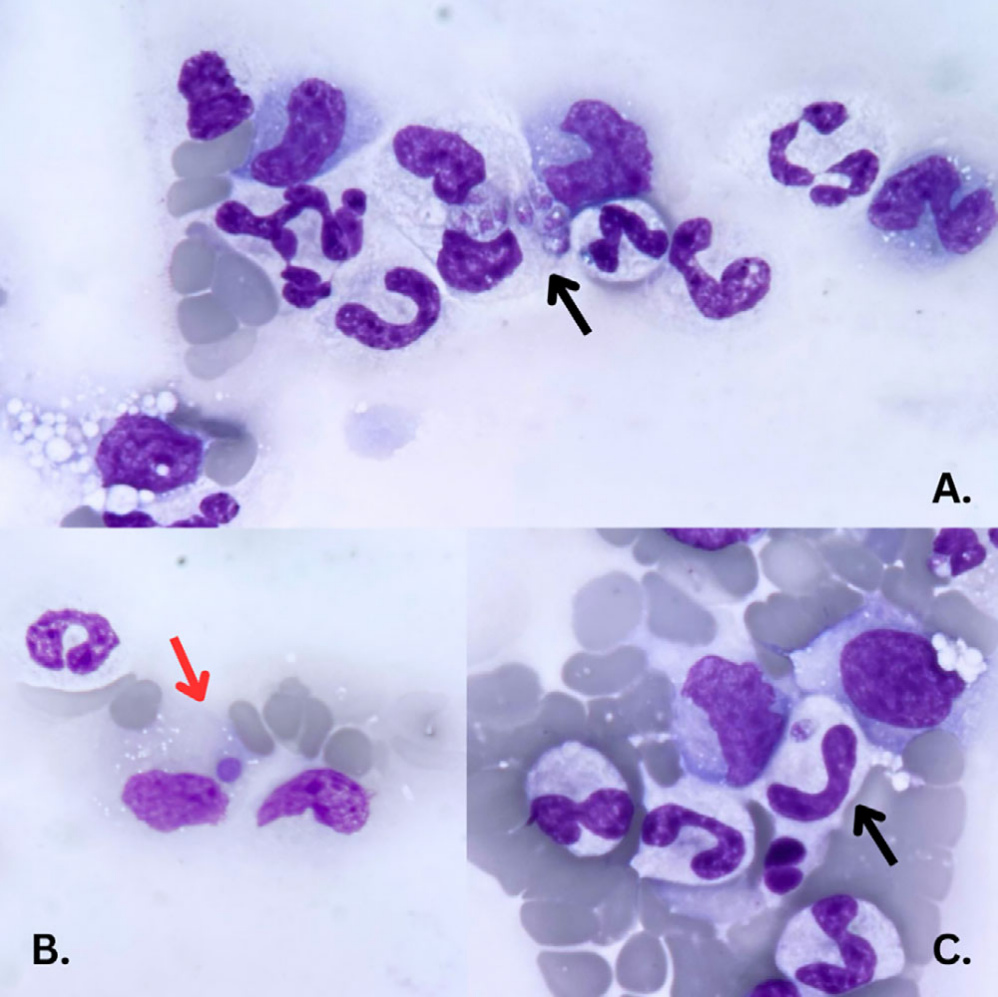
A peripheral blood smear from a dog with systemic toxoplasmosis and canine monocytic ehrlichiosis reveals intracytoplasmic protozoal inclusions (black arrows) consistent with *Toxoplasma gondii* (**A and C**) in activated monocytes and neutrophils, as well as intracytoplasmic morulae of *E. canis* within monocytes (**B**) (red arrow). MGG rapid stain, 100× objective.

A complete blood count (CBC) was performed using the Procyte Dx analyzer (IDEXX Laboratories, Inc., One IDEXX Drive, Westbrook, Maine, USA) and revealed severe normocytic, normochromic and non‐regenerative anaemia with a haematocrit of 11.5% (reference interval [RI]: 37.3%–61.7%). Reticulocytes were substantially below the reference range (28.1 × 10^3^ cells/µL; RI 10–110 × 10^3^). The white blood cell count was within reference ranges, and the haematologic analyzer flagged abnormalities. Severe thrombocytopenia was noted, with a platelet count of 38 × 10^3^ cells/µL (RI: 148 ‐ 484×10^3^ cells/µL) and a mildly increased mean platelet volume (MPV) of 15.1 fL (RI:.7–13.2 fL). A blood smear and buffy coat were done and evaluated. Severe normocytic, normochromic, non‐regenerative anaemia and marked thrombocytopenia were confirmed. Approximately 5% of neutrophils contained multiple (1–3) round to oval to ‘banana’‐shaped structures, approximately 5 µm in length and 1 µm in width, resembling tachyzoites (Figure 1A,C). These structures exhibited pale purple cytoplasm and eccentrically located round pink nuclei, suggesting a protozoal infection. The primary differential diagnoses for the inclusions observed within neutrophils included protozoal infections like *Toxoplasma gondii*, *Neospora caninum or Sarcocystis* spp. (Adkesson et al. 2007). In addition, within approximately 10% of monocytes, granular basophilic microcolonies (morulae) morphologically consistent with *E. canis* were observed (Figure 1B **)**.

Serum biochemistry performed on Catalyst One analyzer (IDEXX Laboratories, Inc., One IDEXX Drive, Westbrook, Maine, USA) revealed moderate hypoglycaemia (47 mg/dL; RI: 72–143 mg/dL). Creatinine was mildly decreased (0.4 mg/dL; RI: 0.5–1.8 mg/dL); although there was mild azotaemia (blood urea nitrogen: 32 mg/dL; RI: 7–27 mg/dL). There was marked hypoalbuminemia (1.4 g/dL; RI: 2.3–4.0 g/dL) but mild hyperglobulinemia (4.7 g/dL; RI: 2.5–4.5 g/dL), causing a marked decrease in A/G ratio to 0.3. There were moderate‐to‐marked increases in hepatocellular enzymes, as follows: ALT (721 U/L; RI: 10–125 U/L), ALP (1609 U/L; RI: 23–212 U/L), and total bilirubin (5.0 mg/dL; RI: 0.0–0.9 mg/dL), with a mild increase in hepatobiliary GGT (16 U/L; RI: 0–11 U/L). Based on the combined CBC and biochemistry findings, an infectious process accompanied by muscle wasting and liver disease was concluded.

**FIGURE 2:**
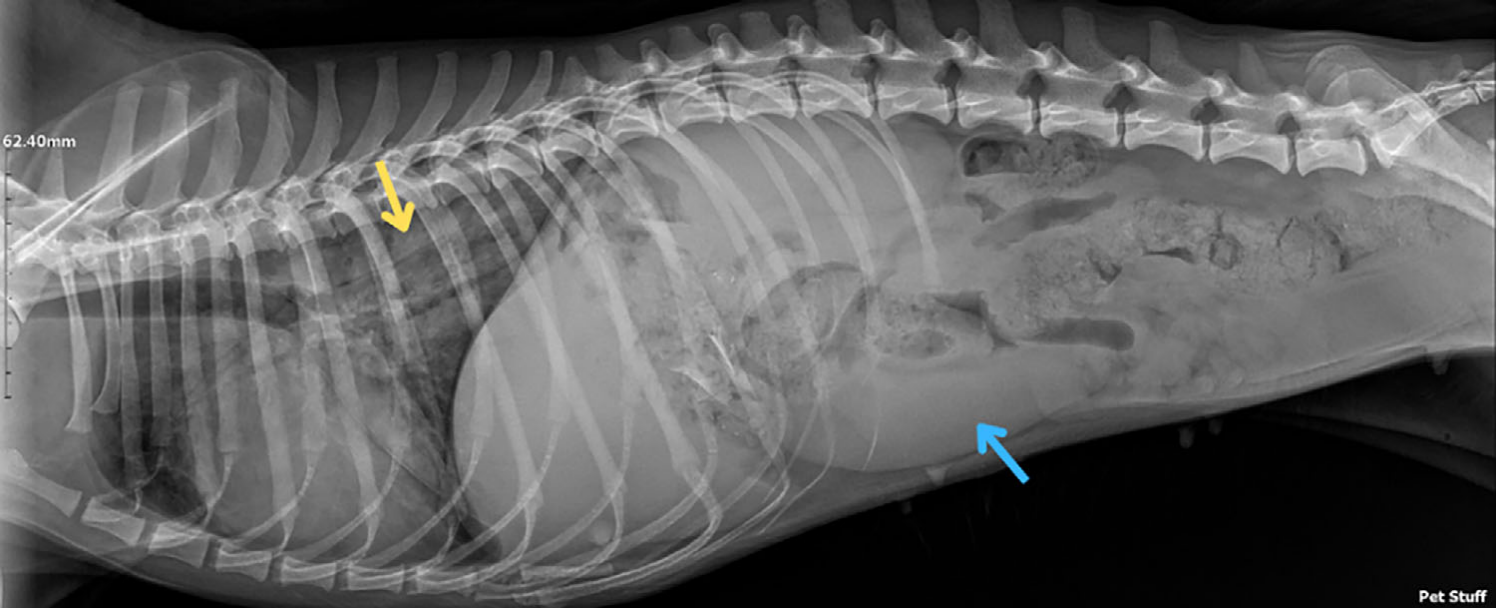
The right lateral view of the thorax displays a diffuse interstitial pattern, predominantly affecting the caudal lung lobes (yellow arrow). Marked splenomegaly is noted (blue arrow).

Thoracic and abdominal radiographs were taken based on the respiratory signs, revealing increased pulmonary radiodensity with a predominantly diffuse interstitial pattern, primarily affecting the caudal lung lobes. This radiographic appearance suggested an infiltrative condition, such as infectious bronchopneumonia or pulmonary oedema. In addition, splenomegaly and mineral content (such as bones or foreign bodies) were present in the gastric region (Figure 2).

Given the presence of tachyzoites in peripheral blood smears, IFAT for *N. caninum* was performed (dog's titer < 1:50, cutoff titer of 1:50), with a negative result. In addition, a serum ELISA for *T. gondii* was positive for IgG, with a titer of 1487.80 LE (cutoff titer: < 50), and negative for IgM, with a titer of 2.54 NTU (cutoff titer: < 9). Both serological tests were conducted at an external laboratory (Laboklin, Germany). Real‐time PCR was conducted to detect *T. gondii*, *E. canis* and *N. caninum*, employing a semiquantitative method to estimate pathogen load. In brief, 200 µL of EDTA‐treated blood was subjected to nucleic acid extraction using a customized protocol adapted from the former QIAamp Cador kit (Qiagen, Hilden, Germany), as previously described (Turcitu 2024). For specific DNA detection, the QuantiNova Pathogen+IC kit (Qiagen, Hilden, Germany) was used according to the manufacturer's instructions, in combination with commercially available primers and probes (Omnigen SRL, Romania). Result validation included both an exogenous internal control (to monitor potential amplification inhibition) and an endogenous control targeting a housekeeping gene (to assess extraction and amplification efficiency), based on Turcitu (2024) and adapted from Wernike (2012). PCR analysis was performed on a Q instrument (Quantabio, Beverly, Massachusetts, USA). The assays returned positive results for *E. canis* and *T. gondii*, with comparable Ct values indicating a medium‐to‐low pathogen load (27.88 for *E. canis* and 27.77 for *T. gondii*). *Neospora caninum* testing yielded negative results.

The dog's clinical state worsened rapidly, and the animal died. The owner refused to perform a necropsy.

## 2 Discussion

This clinical case provides valuable insights into the presentation of co‐infection with *T. gondii* and *E. canis*. The detection of parasitic forms in peripheral blood is indicative of extensive haematogenous dissemination, a finding infrequently documented in veterinary literature. It is plausible that the co‐infection contributed to the accelerated clinical decline and ultimately fatal outcome observed in this patient. Co‐infections of *T. gondii* with distemper virus (Moretti et al. 2006; Aguiar et al. 2012; Headley et al. 2013) and concurrent toxoplasmosis and ehrlichiosis in dogs have been documented in Brazil, with these combinations leading to severe clinical signs and poorer prognoses for affected animals (Moretti et al. 2006; Dubey et al. 2020; Pereira et al. 2024). Toxoplasmosis, which causes significant health issues in immunocompromised individuals, shares clinical features with distemper and ehrlichiosis, which can mimic its symptoms (Bernsteen et al. 1999; Pepper et al. 2019). Other studies have reported clinical toxoplasmosis in animal species, with tachyzoites identified by cytology in the cornea (Swinger et al. 2009), skin lesions (Hoffmann et al. 2012) BAL (Pepper et al. 2019), and CSF (Morganti, Rigamonti, Brustenga, et al. 2024), with only one previous instance of tachyzoites found in circulating white blood cells in a red‐necked wallaby (*Macropus rufogriseus*) (Adkesson et al. 2007). All published clinical cases of canine toxoplasmosis are listed in Table S1.

Although acute monocytic ehrlichiosis was first identified in Romanian dogs more than 9 years ago (Morar et al. 2015), no additional clinical cases have been reported. More recent studies (Mircean et al. 2012; Miró et al. 2022; Sandu et al. 2025) have observed an increasing seroprevalence of *E. canis* in Romania, highlighting the need for further research into the clinical aspects of this often under‐recognized vector‐borne disease, possibly making it underreported and underestimated.

In Romania, *T. gondii* infection has been documented in cats (Györke et al. 2024), with genotype II recognized as the dominant strain, aligning with patterns observed in other European countries (Fernández‐Escobar et al. 2022). Oocysts shed by infected cats serve as a significant source of transmission to intermediate hosts, including humans and dogs (Györke et al. 2024). Considering the dog's unrestricted outdoor access, environmental exposure is a likely route of infection. It is also important to acknowledge the possibility of a serological cross‐reactivity with other protozoal infections in dogs, such as *N. caninum*, *Leishmania* spp. and *Trypanosoma cruzi* (Dubey et al. 2020). In this case, negative serology and PCR results for *N. caninum* effectively exclude it as the cause of clinical signs. However, infections by *Leishmania* spp. and *T. cruzi* cannot be completely ruled out.

An acute *E. canis* infection was confirmed by detecting morulae in the blood smear and a positive PCR result. Interestingly, the rapid serological test was negative for antibodies, which could be explained by the fact that it was a recent infection, as antibodies need 7–28 days to develop (Harrus and Waner 2011). Similar to the case reported in Brazil, where clinical toxoplasmosis was believed to be triggered by canine distemper virus (Aguiar et al. 2012), it is plausible that in the present case, latent toxoplasmosis—confirmed serologically—was reactivated due to immunosuppression induced by acute ehrlichiosis. This reactivation likely played a role in the development of the severe clinical signs observed (Moretti et al. 2006, Pena et al. 2014; da Silva et al. 2015).

However, there is still the question of why serological results pointed to chronic toxoplasmosis. IgM antibodies are generally produced within 2–4 weeks following primary exposure and do not last longer than 3 months. Serologic results indicative of active toxoplasmosis include an increased IgM titer, a high IgG titer in paired serum samples, and a rising IgG titer over time (Davidson et al. 2000). A positive IgG result for *T. gondii* indicated prior exposure or infection, supporting the possibility of toxoplasmosis reactivation. Evidence of recent or active *T. gondii* infection can be further supported by elevated IgM titers or a fourfold or greater increase in IgG titers in paired serum samples (Dini et al. 2024). In this case, a fourfold rise in IgG could not be demonstrated as the patient had deceased, and the IgM levels were within the reference range. Further studies are required to determine whether this immunoglobulin shift occurs in canine patients with toxoplasmosis or if co‐infections influence the development of the immune response.

The clinical signs observed in this dog, attributed to CME, included weight loss, lymphadenomegaly, ocular and nasal discharge, and cutaneous petechiae, with splenomegaly also being associated with CME. Neurological symptoms, such as weakness, paralysis and a diffuse interstitial lung pattern, suggested systemic toxoplasmosis. An interstitial lung pattern accompanied by dyspnoea has previously been described in dogs with pulmonary toxoplasmosis (Pepper et al. 2019; Orbell 2021). In this case, BAL was not performed due to the patient's unstable condition. It is also important to note that similar pulmonary imaging findings and respiratory signs can occur in infections with *Angiostrongylus vasorum*, a cardiopulmonary nematode known to cause severe respiratory distress and interstitial pneumonia in dogs (Taulescu et al. 2023). However, in the present case, no evidence supporting angiostrongylosis was found. In addition, liver dysfunction or hepatitis associated with the underlying infectious diseases could not be excluded, as biochemical findings were indicative of hepatic impairment. Although hepatitis secondary to acute ehrlichiosis has been previously documented (Mylonakis et al. 2019), histopathological confirmation was not done in this case. It is also worth noting that *T. gondii* infection may contribute to hepatic involvement, making it another potential cause of the observed liver abnormalities (Morganti et al. 2024).

While the involvement of additional immunosuppressive factors remains uncertain, the co‐occurrence of toxoplasmosis and ehrlichiosis likely exacerbated the clinical signs, particularly the neurological symptoms, leading to a worse prognosis.

## 3 Conclusions

This case report describes a rare and lethal occurrence in Europe of a dog concurrently infected with canine ehrlichiosis and toxoplasmosis, diagnosed through microscopy, serology and confirmed by PCR. Although it is uncommon to observe *T. gondii* tachyzoites and *E. canis* morulae in peripheral blood cells, this case highlights the diagnostic value of traditional laboratory methods. At the same time, it underscores the inherent challenge in detecting circulating developmental stages of *T. gondii*. The presence of parasitic forms in the blood suggests a widespread haematogenous dissemination, an observation rarely reported in veterinary medicine. Most likely, this co‐infection was responsible for the more severe clinical course in this dog, with a rapid deterioration of health and fatal outcome. Through this case report, the authors would like to advocate for the consideration of co‐infections in canine vector‐borne diseases, with a particular focus on endemic regions.

## Author Contributions

**Ioana Sandu**: data procession, writing first draft. **Georgiana Deak**: writing first draft, revision. **Mihai Turcitu**: molecular work. **Peter James O'Brien**: revision. **Viorica Mircean**: revision. All authors participated in the discussion of the results and were involved in correcting and approving the final manuscript.

## Ethics Statement

The authors confirm that they have complied with the journal's ethical policies, as outlined in the author's guidelines. Furthermore, they declare that they adhered to the National Ethical Guidelines regarding Animal Welfare and Care.

## Consent

The owner provided written informed consent, which was signed to authorize using the specified data.

## Conflicts of Interest

The authors declare no conflicts of interest.

### Peer Review

The peer review history for this article is available at https://publons.com/publon/10.1002/vms3.70380

## Supporting information



Supporting Information

## Data Availability

The data supporting the findings of this study can be obtained from the corresponding author upon reasonable request.
