# Supplementary material for: A Severe Clinical Case of Ehrlichia canis and Toxoplasma gondii in a Dog (With the First Morphological Detection of Tachyzoites in Peripheral Blood)
Source: Vet Med Sci. 2025 May 6;11(3):e70380. doi: 10.1002/vms3.70380 (PMC12055448; doi:10.1002/vms3.70380)
Supplement: Supplementary file 1 — Supporting Information [file VMS3-11-e70380-s001.docx]

| Criteria | Country | Pathogen 1 | Pathogen 2 | Pathogen 3 | Pathogen 4 | Pathogen 5 | Ref |
| --- | --- | --- | --- | --- | --- | --- | --- |
| 1 | Argentina |  |  |  |  | *T. gondii* | Castillo, V. A., Gámbaro, G., & Sinatra, V. (2006). Toxoplasmosis as a cause of subacute thyroiditis in dogs (La toxoplasmosis como causa de tiroiditis subaguda en el perro). *Revista Electrónica de Veterinaria REDVET*, *7*(3). Retrieved from [http://www.veterinaria.org/revistas/redvet/n030306.html](https://mc.manuscriptcentral.com/vetmedsci?DOWNLOAD=TRUE&PARAMS=xik_mkPr54woZaRBBHAwYUkU6tMRfeYG8ukCjQvGn27RgZvkUgeQyWgYirpmCM8SfQgcHvWZbfVPtQnpsrDeFrsLzxCDinVHRYG6tbeh2yQBTpzmsCe7uGmtEb9VqEEfNLugTXWYW8YYX9taLWkfrwxBhvFx2Da3vWamNCPkj36EZXyjtiFtcJSYKbhtvVmSJYdRGSWri6P7mHt2jqM8JzwgLc2oQ691kjrjQrqbAVEJrN8Wj8AuQpQZ9hwbJEKWAKATn5m81nKCFGhahxs2EY467fp3DsyN) |
| 2 | Argentina | CDV |  |  |  | *T.gondii* | Marchetti, C., Grisolia-Romero, M., Zurbriggen, G., Calle, M., Villada, E., & Faya, M. (2023). Canine *Toxoplasma gondii* and distemper virus co-infection in a dog with neuro-ophthalmologic signs from Córdoba, Argentina. *Journal of Clinical & Medical Surgery, 3*(Case Report). Retrieved from [http://www.jclinmedsurgery.com](http://www.jclinmedsurgery.com/) |
| 3 | Australia (7 dogs) |  |  |  | *N.caninum* | *T. gondii* | Al-Qassab S, Reichel MP, Su C, Jenkins D, Hall C, Windsor PA, et al. Isolation of *Toxoplasma gondii* from the brain of a dog in Australia and its biological and molecular characterization. Vet Parasitol. (2009) 164:335–9. doi: 10.1016/j.vetpar.2009.05.019 |
| 4 | Australia |  |  |  |  | *T. gondii* | Anastassiadis, Z., Cook, E., Thompson, A., Herndon, A., McGhie, J., & Meler, E. (2017). Canine systemic toxoplasmosis in south east Queensland: A retrospective case series. *Science Week of the Australian and New Zealand College of Veterinary Scientists*, Gold Coast, QLD, Australia, 6–8 July 2017. |
| 5 | Australia |  |  |  |  | *T. gondii* | Winter, H. (1958). Pathological study of a case of canine toxoplasmosis. *Australian Veterinary Journal, 34*(4), 179–185.<https://doi.org/10.1111/j.1751-0813.1958.tb05865.x> |
| 6 | Australia |  |  |  |  | *T. gondii* | [Conference 05 - 2014 Case: 01 20141001](https://www.askjpc.org/wsco/wsc_showcase2.php?PARAMS=xik_3B6vKBjtE9Rur3JcDnqrHmWXC6RBd8qU4FMiHuPubcfQDhDxBimZWZzcNQBPqSqgD6qoSQYQPpV6vjqboL7DLShC) |
| 7 | Australia |  |  |  |  | *T. gondii* | Pepper, A., Mansfield, C., Stent, A., & Johnstone, T. (2019). Toxoplasmosis as a cause of life-threatening respiratory distress in a dog receiving immunosuppressive therapy. Clinical Case Reports, 7(5), 942–948. https://doi.org/10.1002/ccr3.2121 |
| 8 | Brazil | CDV |  |  |  | *T. gondii* | Aguiar, D. M., Amude, A. M., Santos, L. G. F., Ribeiro, M. G., Ueno, T. E. H., Megid, J., Paes, A. C., Alfieri, A. F., Alfieri, A. A., & Gennari, S. M. (2012). Canine distemper virus and *Toxoplasma gondii* co-infection in dogs with neurological signs. *Arquivo Brasileiro de Medicina Veterinária e Zootecnia, 64*(1).<https://doi.org/10.1590/S0102-09352012000100032> |
| 9 | Brazil |  |  |  |  | *T. gondii* | Pereira, I. P., Gomes, L. F. F., Vieira, T. B., Drummond, F. N., Silva, A. E. L., & Azarias, S. E. (2024). Toxoplasmose canina: Relato de caso [Canine toxoplasmosis: A case report]. *Scientific Electronic Archives*, *17*(6).<https://doi.org/10.36560/17620241981> |
| 10 | Brazil |  |  |  |  | *T. gondii* | Pena HF, Moroz LR, Sozigan RK, Ajzenberg D, Carvalho FR, Mota CM, et al. Isolation and biological and molecular characterization of *Toxoplasma gondii* from canine cutaneous toxoplasmosis in Brazil. J Clin Microbiol. (2014) 52:4419–20. doi: 10.1128/JCM.02001-14 |
| 11 | Brazil) | CDV | CAdV1 | CPV 2 | CAdV-2 | *T. gondii* | Headley et al. Concomitant canine distemper, infectious canine hepatitis, canine parvoviral enteritis, canine infectious tracheobronchitis, and toxoplasmosis in a puppy. J Vet Diagn Invest. (2013) 25:129–35. doi: 10.1177/1040638712471344 |
| 12 | Brazil  (34/111 dogs) |  |  |  |  | *T.gondii* | da Silva AV, Pezerico SB, de Lima VY, d’Arc Moretti L, Pinheiro JP, Tanaka EM, et al. Genotyping of *Toxoplasma gondii* strains isolated from dogs with neurological signs. Vet Parasitol. (2005) 127:23–7. doi: 10.1016/j.vetpar.2004.08.020 |
| 13 | Brazil | CDV |  |  | *E.canis* | *T. gondii* | Moretti Ld, Da Silva AV, Ribeiro MG, Paes AC, Langoni H. *Toxoplasma gondii* genotyping in a dog co-infected with distemper virus and ehrlichiosis rickettsia. Rev Inst Med Trop Sao Paulo. (2006) 48:359–63. doi: 10.1590/S0036-46652006000600012 |
| 14 | Brazil (11/50 dogs) |  |  |  | *N.caninum* | *T. gondii* | Langoni H, Matteucci G, Medici B, Camossi LG, Richini-Pereira VB, Silva RC. Detection and molecular analysis of *Toxoplasma gondii* and *Neospora caninum* from dogs with neurological disorders. Rev Soc Bras Med Trop. (2012) 45:365–8. PMid: 22760137. doi: 10.1590/S0037-86822012000300016 |
| 15 | Brazil |  | *Leishmania braziliensis* |  |  | *T. gondii* | da Silva RC, Caffaro K, Paula CL, Risseti RM, Langoni H, Megid J, et al. An atypical *Toxoplasma gondii* genotype in a rural Brazilian dog co-infected with *Leishmania (Viannia) braziliensis*. Rev Soc Bras Med Trop. (2015) 48:224–7. doi: 10.1590/0037-8682-0284-2014 |
| 16 | Brazil | CDV |  |  | *R. equi* | T.gondii | Portilho, F.V.R., Paes, A.C., Megid, J., Hataka, A., Neto, R.T., Headley, S.A., Oliveira, T.E.S., Colhado, B.S., de Paula, C.L., Guerra, S.T., Mota, A.R., Listoni, F.J.P., Takai, S., Ribeiro, M.G., 2019. Rhodococcus equi pVAPN type causing pneumonia in a dog coinfected with canine morbillivirus (distemper virus) and Toxoplasma gondii. Microb.756 Pathog. 129, 112-117 |
| 17 | Canada |  |  |  |  | *T. gondii* | Wolfer J, Grahn B. Diagnostic ophtalmology. case report of anterior uveitis and endophtalmitis. Can Vet J. (1996) 37:506 |
| 18 | Canada |  |  |  |  | *T. gondii* | Webb JA, Keller SL, Southorn EP, Armstrong J, Allen DG, Peregrine AS, et al. Cutaneous manifestations of disseminated toxoplasmosis in an immunosuppressed dog. J Am Anim Hosp Assoc. (2005) 41:198–202. doi: 10.5326/0410198 |
| 19 | Ecuador |  |  |  |  | *T. gondii* | Villamarín Barragán, D. R., González Salas, R., Vidal del Río, M. M., & Labrada Ching, J. (2023). Toxoplasmosis in jungle dog (*Speothos venaticus*): Case study. *Salud, Ciencia y Tecnología - Serie de Conferencias*, 2023, 737.<https://doi.org/10.56294/sctconf2023737> |
| 20 | France |  |  |  | *Alternaria* sp. | *T. gondii* | Blaga, R., Fabres, V., Leynaud, V., Fontaine, J.-J., Reyes-Gomez, E., Briand, A., Crosaz, O., Lagrange, I., Blaizot, A., Le Roux, D., & others. (2023). *Toxoplasma gondii* and *Alternaria sp.*: An original association in an immunosuppressed dog with persistent skin lesions. *Pathogens, 12*(1), Article 114.<https://doi.org/10.3390/pathogens12010114> |
| 21 | Italy |  |  |  |  | *T. gondii* | Migliore S, La Marca S, Stabile C, Di Marco Lo Presti V, Vitale M. A rare case of acute toxoplasmosis in a stray dog due to infection of *T. gondii* clonal type I: public health concern in urban settings with stray animals? BMC Vet Res. (2017) 13:249. doi: 10.1186/s12917-017-1176-3 |
| 22 | Italy |  |  |  |  | *T. gondii* | Papini R, Mancianti F, Saccardi E. Noise sensitivity in a dog with toxoplasmosis. Vet Rec.(2009) 165:62. doi: 10.1136/vetrec.165.2.62-b |
| 23 | Iraq |  |  |  |  | *T. gondii* | Machattie, C. (1938). Notes on two cases of naturally occurring toxoplasmosis of the dog in Baghdad. *Transactions of The Royal Society of Tropical Medicine and Hygiene, 32*(2), 273–276.<https://doi.org/10.1016/S0035-9203(38)90075-6> |
| 24 | New Zealand |  |  |  | *N.caninum* | *T. gondii* | Patitucci AN, Alley MR, Jones BR, Charleston WAG. Protozoa1 encephalomyelitis of dogs involving *Neosporum caninum* and *Toxoplasma gondii* in New Zealand. N Z Vet J. (1997) 45:231–5. doi: 10.1080/00480169.1997.36035 |
| 25 | New Zealand |  |  |  |  | *T. gondii* | Orbell, G. (2021). Pulmonary toxoplasmosis in a puppy. *In the Lab*. Retrieved from<https://www.awanuivets.co.nz/wp-content/uploads/2021/10/In-the-lab_-Pulmonary-toxoplasmosis-in-a-puppy.pdf> |
| 26 | Panama |  |  |  |  | *T. gondii* | Grocott, R. G. (1950). A case of canine toxoplasmosis from the Canal Zone. *The American Journal of Tropical Medicine and Hygiene, 30*(s1-5), 669–675.<https://doi.org/10.4269/ajtmh.1950.s1-30.669> |
| 27 | South Africa |  |  |  |  | *T. gondii* | Smit, J. D. (1961). Toxoplasmosis in dogs in South Africa: Seven case reports. *South African Veterinary Medical Association Journal*, *32*(3).<https://hdl.handle.net/10520/AJA00382809_904> |
| 28 | Singapore |  |  |  |  | *T. gondii* | Chen, A., Boulay, M., Chong, S., & others. (2023). Suspected clinical toxoplasmosis in a 12-week-old puppy in Singapore. *BMC Veterinary Research, 19*, 110.<https://doi.org/10.1186/s12917-023-03674-5> |
| 29 | Uruguay |  |  |  |  | *T. gondii* | Dorsch, M. A., Cesar, D., Bullock, H. A., Uzal, F. A., Ritter, J. M., & Giannitti, F. (2022). Fatal *Toxoplasma gondii* myocarditis in an urban pet dog. *Veterinary Parasitology: Regional Studies and Reports, 27*, 100659.<https://doi.org/10.1016/j.vprsr.2021.100659> |
| 30 | USA |  |  |  |  | *T. gondii* | Swinger RL, Schmidt KA Jr, Dubielzig RR. Keratoconjunctivitis associated with Toxoplasma gondii in a dog. Vet Ophthalmol. (2009) 12:56–60. doi: 10.1111/j.1463-5224.2009.00675.x |
| 31 | USA |  |  |  |  | *T. gondii* | Bernsteen L, Gregory CR, Aronson LR, Lirtzman RA, Brummer DG. Acute toxoplasmosis following renal transplantation in three cats and a dog. J Am Vet Med Assoc. (1999) 215:1123–6. |
| 32 | USA |  |  |  |  | *T. gondii* | Hoffmann AR, Cadieu J, Kiupel M, Lim A, Bolin SR, Mansell J. Cutaneous toxoplasmosis in two dogs. J Vet Diagn Invest. (2012) 24:636–40. doi: 10.1177/1040638712440995 |
| 33 | USA |  |  |  |  | *T. gondii* | Vallone, L. V., Johnson, M. C., Guo, F., & Zhu, G. (2018). What is your diagnosis? Corneal scrape from a dog. *Veterinary Clinical Pathology, 47*(2), 315–316.<https://doi.org/10.1111/vcp.12587> |
| 34 | USA |  |  |  |  | T. gondii | Schlemmer, F., Dubey, J. P., & Gangadhar, R. (2018). Toxoplasma gondii and ocular manifestations in dogs. Veterinary Ophthalmology, 21(3), 226–235. https://doi.org/10.1111/vop.12493 |
